# Clinical features combined with ultrasound characteristics to predict TERT promoter mutations in papillary thyroid carcinoma: a single-center study over the past 5 years

**DOI:** 10.3389/fendo.2024.1322731

**Published:** 2024-03-18

**Authors:** Yan Hu, Shangyan Xu, Lei Dong, Zuxian Pan, Lu Zhang, Weiwei Zhan

**Affiliations:** ^1^ Department of Ultrasound, Ruijin Hospital, Shanghai Jiaotong University School of Medicine, Shanghai, China; ^2^ Department of Pathology, Ruijin Hospital, Shanghai Jiaotong University School of Medicine, Shanghai, China

**Keywords:** papillary thyroid cancer, thyroid nodule, TERT, ultrasound, molecular marker

## Abstract

**Purpose:**

Telomerase reverse transcriptase (TERT) has been reported in papillary thyroid carcinoma (PTC). This study aimed to investigate the correlation of TERT promoter mutations with clinical and ultrasound (US) features in PTC and to develop a model to predict TERT promoter mutations.

**Methods:**

Preoperative US images, postoperative pathological features, and TERT promoter mutation information were evaluated in 365 PTC patients confirmed by surgery. Univariate and multivariate factor analyses were performed to identify risk factors for TERT promoter mutations. A predictive model was established to assess the clinical predictive value.

**Results:**

Of the 365 patients with PTC (498 nodules), the number of those with TERT promoter mutations was 67 cases (75 nodules), and the number of those without mutations was 298 cases (423 nodules). The median age was 40 years in the wild-type group and 60 years in the mutant group. Male patients made up 35.82% of the mutant group and 22.82% of the wild-type group. Multivariate analysis revealed that the independent risk factors associated with the occurrence of TERT promoter mutation in PTC were as follows: older age (odds ratio (OR) = 1.07; *p* = 0.002), maximum diameter of ≥ 10 mm (OR = 3.94; *p* < 0.0001), unilateral (OR = 4.15; *p* < 0.0001), multifocal (OR = 7.69; *p* < 0.0001), adjacent to the thyroid capsule (OR = 1.94; *p* = 0.044), and accompanied by other benign nodules (OR = 1.94, *p* = 0.039). A predictive model was established, and the area under the curve (AUC) of the receiver operating characteristic was 0.839. TERT promoter mutations were associated with high-risk US and clinical features compared with the wild-type group.

**Conclusion:**

TERT promoter mutations were associated with older ages. They were also found to be multifocal, with a maximum diameter of ≥ 10 mm, unilateral, adjacent to the thyroid capsule, and accompanied by other benign nodules. The predictive model was of high diagnostic value.

## Introduction

Thyroid cancer (TC) is the ninth most prevalent cancer worldwide, with its incidence has gradually increased in recent years (1, 2). Among all types of TC, papillary thyroid cancer (PTC) is the most common and shows an increasing trend in all regions, despite wide regional variations (3). With further studies on the pathogenesis of TC, similar to other cancer types, TC occurs and develops through the gradual accumulation of various genetic and epigenetic alterations (4, 5). Recent advances in the genetic characterization of TC have provided molecular markers for adjuvant diagnostic and therapeutic targets (6–8).

Telomerase reverse transcriptase (TERT) promoter mutations are most commonly observed in malignant melanoma, uroepithelial bladder cancer, glioblastoma, mucinous liposarcoma, as well as in certain skin cancer and medulloblastoma subtypes. The TERT promoter mutation rate in these cancers can reach 80%–90%, with intermediate rates of 10%–50% in TC (9). TERT promoter mutations occur mainly in two hotspots on chromosome 5 (1,295,228 and 1,295,250, or −124 bp and −146 bp of the ATG) with cytidine to thymidine (C>T) dipyrimidine transitions, known as C228T and C250T, respectively (10, 11).

Previous studies have found a significant correlation between TERT promoter mutations and distant metastases, higher pathological stage, disease recurrence, disease-specific mortality, and other adverse prognostic features in 647 differentiated thyroid carcinoma lesions, especially in PTC (12). These have also been confirmed in other studies (13–17). Therefore, in the American Association of Endocrine Surgeons Guidelines for the Definitive Surgical Management of Thyroid Disease in Adults, there is an acknowledgment of the inclusion of the TERT promoter mutation in the assessment of the overall mutational burden in thyroid cancers (18). At present, clinical testing for mutations in the TERT promoter is usually done by performing Fine needle aspiration (FNA) on the target nodule, but the cost of testing is a burden for patients. This study aims to adopt a simpler, more economical, and noninvasive approach to predict TERT promoter mutations and to assist in screening high-risk PTC populations with adverse prognostic features such as a high recurrence rate and mortality rate in order to adopt corresponding clinical management strategies timely.

In this study, we propose to compare the differences in clinical and ultrasound (US) features between the TERT promoter mutation group and the wild-type group. Subsequently, to predict TERT promoter mutations in PTC, we will combine clinical features and US characteristics to construct a predictive model. It will provide a noninvasive way to identify high-risk individuals, enabling doctors to tailor personalized treatments and monitoring strategies promptly.

## Materials and methods

### Patients

This was a retrospective study that was reviewed and approved by the Ethics Review Committee of Ruijin Hospital, Shanghai Jiaotong University School of Medicine, and the requirement for obtaining informed consent from patients was waived because of its retrospective nature. We reviewed 365 patients who were diagnosed with primary PTC and underwent surgery at Ruijin Hospital between Jane 2018 and June 2023; they were enrolled in the present study. Molecular testing for TERT promoter mutations was performed on all PTC cases. Clinicopathologic information was retrieved from electronic medical records. The inclusion criteria were as follows: (1) all underwent surgery treatment with a pathologically confirmed diagnosis of PTC; (2) complete postoperative pathological tissue specimens were available; and (3) complete records of TERT promoter testing were available. The exclusion criteria include the following: (1) incomplete US image data and (2) minor patients under 18 years old.

### Clinical, US, and pathology assessment

All patients’ clinical characteristic information, US images, and pathology results are from the HIS system. All grayscale and Doppler sonographic examinations were performed with a 4- to 13-MHz linear probe (MyLab 90, EsaoteSpA, Genoa, Italy; iU22 System, Philips, Seattle, WA, USA; and Resona 7, Mindray, Shenzhen, China) by two radiologists with more than 10 years of experience in thyroid US. Doppler parameters were optimized to maximize Doppler sensitivity. Adjacent to the thyroid capsule refers to the distance between the thyroid nodules and the thyroid capsule, which is less than 2 mm. Accompanied by other benign nodules means that, in addition to the malignant nodules, there are other nodules categorized as TI-RADS 2 to TI-RADS 3 in the same patients. Cervical lymph node metastasis was detected via US. The reference ranges for Thyroid Stimulating Hormone (TSH), Thyroglobulin (Tg), Thyroid peroxidase antibody (TPOab), anti-thyroglobulin antibodies (Tgab), and calcitonin are as follows: TSH ranges from 0.27 µIU/mL to 4.2 µIU/mL, Tg ranges from 3.5 ng/mL to 77 ng/mL, TPOab ranges from 0 IU/mL to 34 IU/mL, Tgab ranges from 0 IU/mL to 115 IU/mL, and calcitonin ranges from 0 pg/mL to 6.4 pg/mL. The determination of histological diagnosis is based on the criteria and terminology proposed by the World Health Organization.

### Detection of TERT promoter mutations

For each tumor, a tissue block with the most representative tumor area and high tumor cell enrichment (tumor cell purity > 50%) was selected for amplification-refractory mutation system PCR (ARMS-PCR). Tumor cell areas were labeled and macroscopically dissected to detect tumor purity. ARMS-PCR was performed for the TERT promoter mutations, as described previously (19, 20). TERT promoter mutant DNA is detected by the TERT gene mutation kit (Amoydx, Shanghai, China), according to the manufacturer’s instructions. ARMS-PCR was performed on Stratagene Mx3000P™ (Stratagene, USA) and following the standard procedures: initial template denaturation at 95°C for 5 min was followed by 15 cycles of 95°C for 25 s, 64°C for 20 s, and 72°C for 20 s, and 31 cycles of 93°C for 25 s, 60°C for 35 s, and 72°C for 20 s. FAM and HEX signals were collected at 60°C to perform real-time PCR. Positive values and results are interpreted according to the kit instructions: when the CT value is greater than or equal to the negative threshold Ct value (28 or 29), it is regarded as negative; when the mutant Ct value of the sample is less than the negative threshold Ct value and is less than 26, it is regarded as positive. When the Ct value ranges from 26 to the negative threshold, it is necessary to combine it with the ΔCt cut-off value to determine the positive value.

### Statistical analysis

Categorical variables were compared using the Pearson’s Chi-square test or Fisher’s exact test based on TERT promoter mutation status. Independent samples *t*-test or Mann–Whitney *U* test was used to compare continuous variables. Univariate and multivariate logistic regression analyses were performed to identify clinical features and US associated with TERT promoter mutation status. Results were considered statistically significant with a two-tailed *p*-value of less than 0.05. All data were analyzed using SPSS v.22.0 software (IBM Corp.), STATA 16 (Stata Corp.), and R version 3.6.3 (http://www.r-project.org/).

## Results

### Clinical and US characteristics of individual patient

A total of 365 PTC patients confirmed by pathology were included for analysis of clinical and ultrasound risk factors associated with TERT promoter mutations. Of these, 67 were in the mutant group and 298 in the wild-type group. It showed differences in median age (40 years vs. 60 years; *p* < 0.05) and in the gender distribution (percentage of men, 22.82% vs. 35.82%; *p* < 0.05) at the time of consultation. Furthermore, the two groups exhibit significant differences in TSH, Tgab, Tpoab, Tg, cervical lymph node metastasis in the lateral neck region, and the number of malignant thyroid nodules between the two groups. However, there were no significant differences in calcitonin, family history, cervical lymph node metastasis, and cervical lymph node metastasis in the central region (**Table 1**).

**Table 1:** Clinical and US characteristics of individual patients.

Features	TERT wt (*n* = 298)		TERT mut (*n* = 67)		*p*-value
Age (year)	40 (32–53)		60 (50–70)		0.0001
Sex					
Male	68	22.82%	24	35.82%	0.027
Female	230	77.18%	43	64.18%	
TSH					
Low	0	0	8	11.94%	< 0.0001
Normal	291	97.65%	59	88.06%	
High	7	2.35%	0	0	
Tgab					
Normal	256	85.91%	67	100%	0.001
High	42	14.09%	0	0	
Tpoab					
Normal	214	71.81%	66	98.51%	< 0.0001
High	84	28.19%	1	1.49%	
Tg					
Low	19	6.38%	66	98.51%	< 0.0001
Normal	236	79.19%	1	1.49%	
High	43	14.43%	0	0.00%	
Calcitonin					
Normal	291	97.65%	67	100%	0.205
High	7	2.35%	0	0.00%	
Family history					
No	265	88.93%	60	89.55%	0.98
TN	16	5.37%	4	5.97%	
TC	4	1.34%	1	1.49%	
TFD	1	0.34%	0	0.00%	
OC	12	4.03%	2	2.99%	
CLNM					
No	194	65.10%	43	64.18%	0.886
Yes	104	34.90%	24	35.82%	
cCLNM					
No	221	74.16%	52	77.61%	0.557
Yes	77	25.84%	15	22.39%	
lCLNM					
No	255	85.57%	50	74.63%	0.029
Yes	43	14.43%	17	25.37%	
NMTN					
1	205	68.79%	63	94.03%	< 0.001
2	69	23.15%	2	2.99%	
≥ 3	24	8.05%	2	2.99%	

TERT wt, TERT promoter wild type; mut, TERT promoter mutation; TN, thyroid nodules; TC, thyroid cancers; TFD, thyroid function disorder; OC, other cancers; CLNM, cervical lymph node metastasis; cCLNM, cervical lymph node metastasis in the central region; lCLNM, cervical lymph node metastasis in the lateral neck region; NMTN, number of malignant thyroid nodules.

### US characteristics of nodules

A total of 498 nodules were included to analyze the relation between US characterization of nodules and TERT promoter mutations. Nodules with TERT promoter wild-type (*n* = 423) and TERT promoter mutation (*n* = 75) showed differences in the following nine characteristics: maximum diameter of ≥ 10 mm, unilateral, multifocal, markedly hypoechoic, irregular margin, absence of microcalcification, rich blood supply, adjacent to the thyroid capsule, and accompanied by other benign nodules. In contrast, there were no significant differences in terms of location in the left, right, or isthmus (*p* = 0.760) and vertical orientation (*p* = 0.206) (**Table 2**).

**Table 2:** US characteristics of thyroid nodules.

Characteristics	TERT wt (*n* = 423)		TERT mut (*n* = 75)		*p*-value
MD (mm)					
< 10	262	61.94%	23	30.67%	< 0.0001
≥ 10	161	38.06%	52	69.33%	
Location					
Left	190	44.92%	37	49.33%	0.76
Right	212	50.12%	35	46.67%	
Isthmus	21	4.96%	3	4.00%	
Unilateral					
No	155	36.64%	13	17.33%	0.001
Yes	268	63.36%	62	82.67%	
Echo					
Markedly hypoechoic	3	0.71%	3	4.00%	0.033
Hypoechoic	414	97.87%	72	96.00%	< 0.0001
Others	6	1.42%	0	0.00%	
Multifocal					
No	188	44.44%	13	17.33%	< 0.0001
Yes	235	55.56%	62	82.67%	
Vertical orientation					
No	209	49.41%	43	57.33%	0.206
Yes	214	50.59%	32	42.67%	
Irregular margin					
No	81	19.15%	7	9.33%	0.04
Yes	342	80.85%	68	90.67%	
Echogenic foci					
No	127	30.02%	28	37.33%	0.028
MC	262	61.94%	47	62.67%	
Others	34	8.04%	0	0.00%	
Blood supply					
No	18	4.26%	0	0.00%	0.011
Poor	323	76.36%	53	70.67%	
Rich	82	19.39%	22	29.33%	
ATC					
No	205	48.58%	27	36.00%	0.044
Yes	217	51.42%	48	64.00%	
OBN					
No	196	47.13%	21	28.00%	0.002
Yes	221	52.87%	54	82.00%	

MD, maximum diameter; MC, microcalcifications; ATC, adjacent to the thyroid capsule; OBN, accompanied by other benign nodules.

### Lasso regression and multivariate analysis

**Figure 1:**
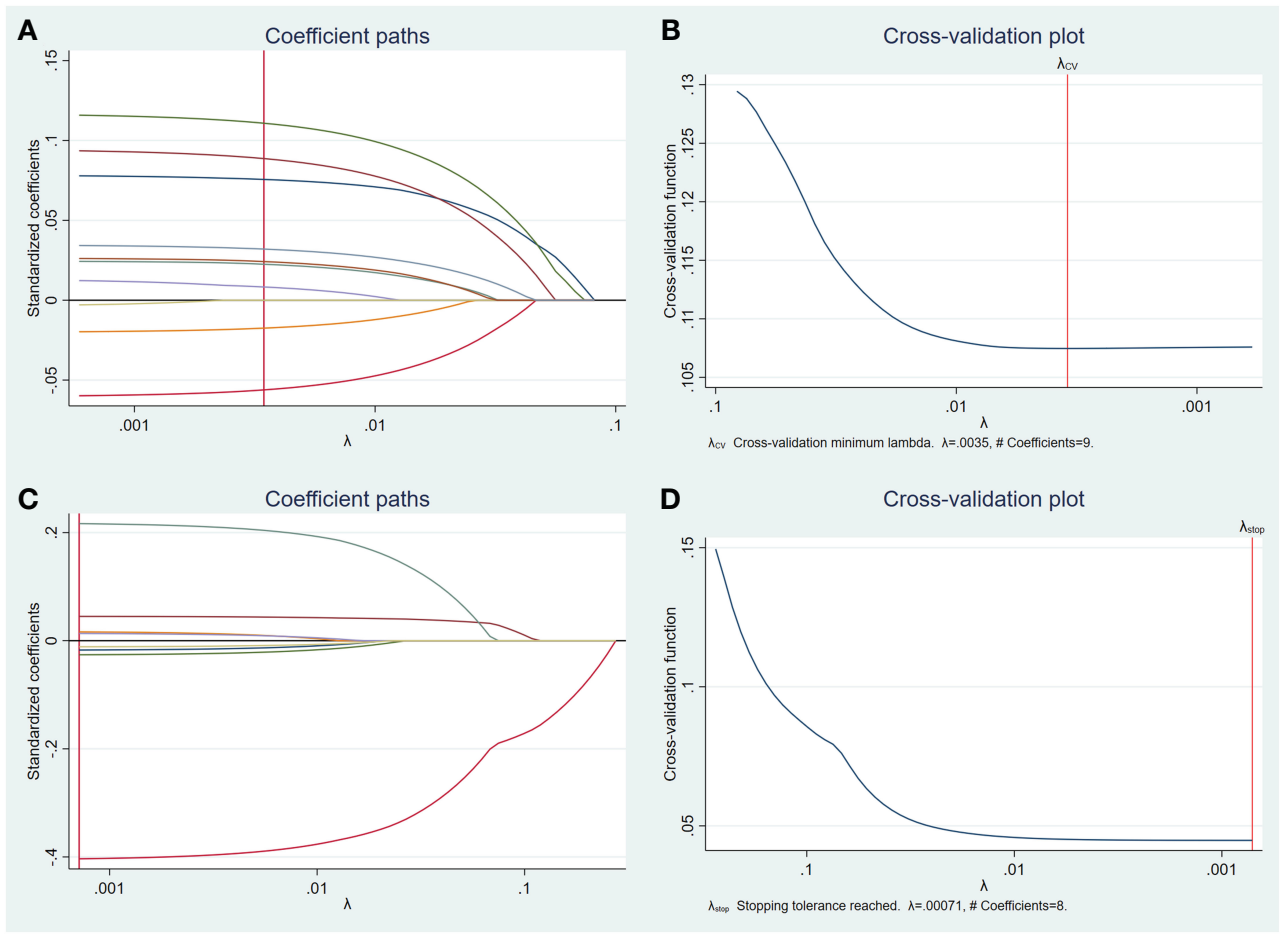
Lasso regression for risk factors from univariate analysis. Lasso regression screening was performed on nodule features that showed differences in the results of the preliminary univariate analysis, and the colored lines from right (blue) to left (purple) in order represent the following factors: maximum diameter of ≥ 10 mm, multifocal, unilateral, microcalcification, accompanied by other benign nodules, adjacent to the thyroid capsule, markedly hypoechoic, irregular margin, rich blood supply **(A)**. Selection of the tuning parameter lambda **(B)**. Lasso regression was performed to screen for patient characteristics that showed differences, and the first four colored lines from right (red) to left (green) represent, in order, the following indicators: age, gender, cervical lymph node metastasis in the lateral neck region, and number of malignant thyroid nodules, while others represent TSH, Tgab, Tpoab, and Tg **(C)**. Selection of the tuning parameter lambda **(D)**.

Univariate analysis was used to compare the clinical features and US characteristics of the patients, as well as the US characteristics of the nodules, between the two groups. Preliminary results showed that risk factors associated with the development of TERT promoter mutations in PTC were older age, male, low TSH, normal Tgab and Tpoab, low Tg, cervical lymph node metastasis in the lateral neck region, number of malignant thyroid nodules, maximum diameter of ≥ 10 mm, unilateral, markedly hypoechoic, irregular margin, nonmicrocalcification, rich blood supply, adjacent to the thyroid capsule, and accompanied by other benign nodules. To exclude the effect of multicollinearity, we used lasso 10-fold cross-validation to screen for true risk factors from the candidate variables identified by univariate analysis (**Figure 1**). After correction, risk factors analyzed by univariate analysis were male, older age, number of malignant thyroid nodules, cervical lymph node metastasis in the lateral neck region, maximal diameter of ≥ 10 mm, unilateral, multifocal, markedly hypoechoic, nonmicrocalcification, adjacent to the thyroid capsule, and accompanied by other benign nodules. Further multivariate analysis revealed that the independent risk factors for TERT promoter mutation were older age (OR = 1.07; *p* = 0.002), maximum diameter of ≥ 10 mm (OR = 3.94; *p* < 0.0001), unilateral (OR = 4.15; *p* < 0.0001), multifocal (OR = 7.69; *p* < 0.0001), adjacent to the thyroid capsule (OR = 1.94; *p* = 0.044), and accompanied by other benign nodules (OR = 1.94; *p* = 0.039) (**Tables 3**, **4**).

**Table 3:** Clinical and US feature analyses of patients.

Variant	OR	95% CI	*p*-value
Sex			
Male	Reference		0.059
Female	0.27	0.07–1.05	
Age	1.07	1.03–1.12	0.002
NMTN			
1	Reference		
2	0.46	0.05–4.158	0.492
≥ 3	1.81	0.07–43.92	0.718
lCLNM			
No	Reference		0.440
Yes	1.83	0.39–8.55	

**Table 4:** US feature screening based on multifactor logistic regression.

Variant	OR	95% CI	*p*-value
MD (mm)			
< 10	Reference		< 0.0001
≥ 10	3.94	2.08–7.44	
Unilateral			
No	Reference		< 0.0001
Yes	4.15	2.00–8.64	
Multifocal			
No	Reference		< 0.0001
Yes	7.69	3.74–15.82	
Echo			
Markedly hypoechoic	Reference		0.065
Hypoechoic	0.14	0.02–1.13	
MC			
Yes	Reference		0.052
No	0.51	0.26–1.01	
ATC			
No	Reference		0.044
Yes	1.94	1.02–3.70	
OBN			
No	Reference		0.039
Yes	1.94	1.04–3.64	

### Predictive model

**Figure 2:**
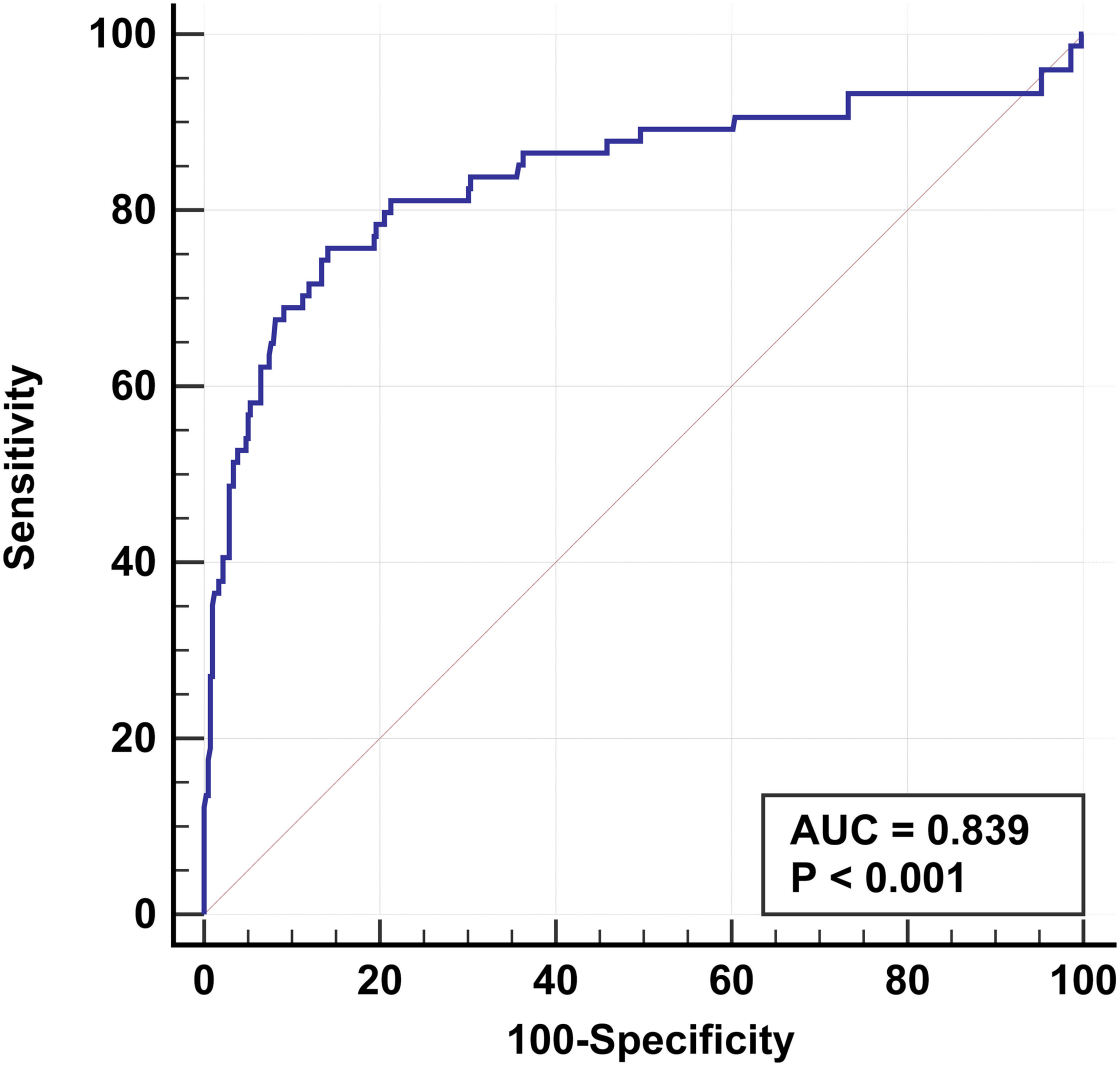
Receiver operating characteristic based on results of multifactorial analysis. The receiver operating characteristics are established for older age, multifocal, maximum diameter of ≥ 10 mm, unilateral, adjacent to the thyroid capsule, and accompanied by other benign nodules. The AUC is 0.839.

Six clinical and US features were identified as independent risk factors for predicting the occurrence of TERT promoter mutations in PTC. These include older age, maximum diameter of ≥ 10 mm, unilateral, multifocal, adjacent to the thyroid capsule, and accompanied by other benign nodules. Based on these factors, a predictive model was established with an area under the curve (AUC) of the receiver operating characteristic of 0.839 (**Figure 2**).

## Discussion

We examined the TERT promoter status in 365 PTC patients confirmed by pathology. The results of the univariate analysis suggest that the TERT promoter mutation may be associated with several factors, including older age, male, low TSH, normal Tgab, normal Tpoab, low Tg, cervical lymph node metastasis in the lateral neck region, number of malignant thyroid nodules, maximum diameter of ≥ 10 mm, unilateral, markedly hypoechoic, irregular margin, nonmicrocalcification, rich blood supply, adjacent to the thyroid capsule, and accompanied by other benign nodules. However, after correction using machine learning and further multivariate analysis, we discovered that older age, maximum diameter of ≥ 10 mm, unilateral, multifocality, adjacent to the thyroid capsule, and accompanied by other benign nodules were independent risk factors for predicting TERT promoter mutation. Finally, we established a prediction model for TERT promoter mutations based on the above independent risk factors. The model achieved an AUC of 0.839, indicating good predictive performance, and may become a promising predictive tool for clinical practice.

Our study showed that for older patients with PTC, the risk of TERT promoter mutation was higher (OR = 1.07; *p* < 0.05), and the risk of TERT mutation increased with each 1-year increase in age, that is, by 7%. Additionally, maximum diameter of ≥ 10 mm, unilateral, multifocal, and adjacent to the thyroid capsule were identified as independent risk factors for TERT promoter mutation in PTC patients. Furthermore, our study also counted and compared the presence of other benign nodules in patients with PTC and found that differences emerged between the two groups. This confirmed that the presence of other benign nodules could serve as an independent risk factor for predicting the occurrence of TERT promoter mutations in PTCs.

It is worth noting that when counting the variables, we recorded the number of malignant thyroid nodules in the PTC patients and compared the multifocality in the ultrasound features of the nodules between the two groups. Although both variables referred to the same thing and showed differences in the initial univariate analysis, the number of malignant thyroid nodules was excluded after eliminating the effect of multicollinearity. The multifocality ultimately remained an independent risk factor for predicting the TERT promoter mutations.

Previous studies have demonstrated that age, maximum diameter of the nodules, multifocality, and adjacent to the thyroid capsule are independent risk factors for TERT promoter mutations, which are consistent with our findings (21–23). Especially age, which has been widely reported in many studies, was consistently validated (24–26). In addition, other studies have suggested that indicators such as males, irregular margins, and vertical orientation can predict TERT promoter mutations (27, 28). In our study, the results of univariate analysis showed a possible relationship between TERT promoter mutations and male and irregular margins. However, these two variables were excluded for multiple covariance. In contrast, vertical orientation did not show significant differences in our study. While unilateral and accompanied by other benign nodules have rarely been analyzed before, even fewer studies have suggested an association with the presence of mutations in the TERT promoter. This study found that these two are independent risk factors for TERT promoter mutations.

A meta-analysis that included 51 studies indicated that cervical lymph node metastasis was associated with TERT promoter mutations, which was additionally confirmed by a meta-analysis that included a total of 2,035 patients from eight studies (29, 30). There is also a study suggesting that cervical lymph node metastasis is not associated with TERT promoter mutations (31). Therefore, the correlation between cervical lymph node metastasis and TERT promoter mutations remains controversial. Our results suggest that cervical lymph node metastasis detected by US was not an independent risk factor for the TERT promoter mutation.

In this study, a predictive model based on relevant clinical and US features can serve as an auxiliary tool for the precise management of PTC patients. The combination of clinical and US features can help predict TERT promoter mutations in a noninvasive way, replacing FNA to reduce patient pain and medical costs. Based on previous research reports and the results of this study, the mutation of the TERT promoter was found to be associated with the adjacent thyroid capsule and adverse prognostic features such as high recurrence rate and mortality rate. Therefore, detection of TERT promoter mutations can assist in rationally selecting appropriate treatment strategies for those high-risk populations, such as establishing more frequent follow-up plans. Conversely, individuals at relatively low risk can opt for more conservative treatment to reduce overtreatment.

There are some limitations to this study. First, the retrospective study design might limit the analysis of additional potential variables. Secondly, the predictive model lacks external validation. Additionally, although the sample size has notably increased compared to prior studies, a larger prospective cohort could provide more robust and persuasive findings. Therefore, future research will prioritize rigorous prospective, multicenter studies that focus on validating and optimizing this predictive model.

In conclusion, our study demonstrated that older age, maximum diameter of ≥ 10 mm, unilateral, multifocal, adjacent to the thyroid capsule, and accompanied by other benign nodules were independent risk factors for TERT promoter mutations in PTC. The prediction model based on these characteristics was of high predictive value. The correct acquisition and interpretation of the patient’s clinical characteristics as well as the US characteristics of the nodule are important and may aid in predicting the TERT promoter status, providing a valuable reference for subsequent patient management and treatment options.

## Data availability statement

The original contributions presented in the study are included in the article/supplementary material. Further inquiries can be directed to the corresponding authors.

## Ethics statement

The studies involving humans were approved by the ethics review committee of Ruijin Hospital, Shanghai Jiaotong University School of Medicine. The studies were conducted in accordance with the local legislation and institutional requirements. The requirement for obtaining informed consent from patients was waived because of its retrospective nature.

## Author contributions

YH: Writing – review & editing, Writing – original draft, Visualization, Validation, Supervision, Software, Resources, Project administration, Methodology, Investigation, Formal analysis, Data curation, Conceptualization. SX: Software, Conceptualization, Writing – review & editing, Visualization, Validation, Supervision, Resources, Project administration, Methodology, Investigation. LD: Methodology, Formal analysis, Writing – review & editing. ZP: Investigation, Writing – review & editing, Resources, Formal analysis, Data curation. LZ: Methodology, Validation, Resources, Writing – review & editing. WZ: Visualization, Validation, Supervision, Project administration, Methodology, Funding acquisition, Writing – review & editing, Resources, Investigation.
